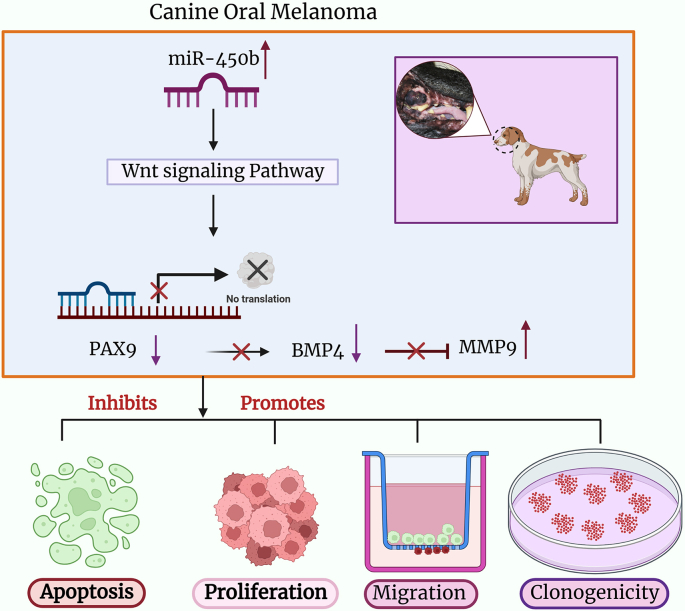# Upregulation and functional roles of miR-450b in canine oral melanoma

**DOI:** 10.1016/j.ncrna.2024.01.017

**Published:** 2024-02-01

**Authors:** MD Nazmul Hasan, Md. Mahfuzur Rahman, Al Asmaul Husna, Mohammad Arif, Indre Jasineviciute, Daiki Kato, Takayuki Nakagawa, Naoki Miura

**Affiliations:** a Joint Graduate School of Veterinary Medicine, Kagoshima University, 1-21-24, Korimoto, Kagoshima, 890-0065, Japan; b Veterinary Teaching Hospital, Joint Faculty of Veterinary Medicine, Kagoshima University, 1-21-24, Korimoto, Kagoshima, 890-0065, Japan; c Department of Human Oncology, University of Wisconsin School of Medicine and Public Health, Madison, WI, USA; d Department of Anatomy and Physiology, Veterinary Faculty, Lithuanian University of Health Sciences, LT-47181, Kaunas, Lithuania; e Laboratory of Veterinary Surgery, Graduate School of Agricultural and Life Sciences, The University of Tokyo, Tokyo, 113-8657, Japan

**Keywords:** miR-450b, Proliferation, Migration, Colony formation, Apoptosis, PAX9-BMP4-MMP9

## Abstract

Canine oral melanoma (COM) is a common and highly aggressive disease with the potential to model human melanomas. Dysregulated microRNAs represent an interesting line of research for COM because they are implicated in tumor progression. One example is miR-450b, which has been investigated for its molecular mechanisms and biological functions in multiple human cancers, but not human or canine melanoma. Here, we aimed to investigate miR-450b as a potential diagnostic biomarker of COM and its functional roles in metastatic and non-metastatic forms of the disease. We investigated the expression of miR-450b and its target mRNA genes in clinical (tumor tissue and plasma) samples and metastatic and primary-tumor cell lines. Knockdown and overexpression experiments were performed to determine the influence of miR-450b on cell proliferation, migration, colony formation, and apoptosis. miR-450b was significantly upregulated in COM and differentiated between metastatic and non-metastatic tumors, and its potential as a biomarker of metastatic and non-metastatic COM was further confirmed in ROC analysis. miR-450b knockdown promoted cell proliferation, migration, and clonogenicity and inhibited apoptosis, whereas its overexpression yielded the reverse pattern. miR-450b directly binds 3’ UTR of PAX9 mRNA and modulates its function leading to BMP4 downregulation and MMP9 upregulation at the transcript level. Furthermore, we surmised that miR-450b activates the Wnt signaling pathway based on gene ontology and enrichment analyses. We concluded that miR-450b has the potential as a diagnostic biomarker and could be a target candidate for COM treatment.

## Abbreviations

CCK-8cell counting kit-8
COM: canine oral melanoma
FC: Fold change
Go: Gene ontology
KEGG ncyclopedia Genes and Genomes, miRNA, microRNA
NC: Negative control
PBS: phosphate-buffered saline.

## 1 Introduction

Oral melanoma is a highly aggressive disease in dogs and humans, associated with a high rate of malignancy and poor prognosis [[1], [2], [3]]. Canine oral melanoma (COM) has a fatality rate exceeding 70 % at phases II-III and is fatal for many dogs within one year of surgery [3]. COM may provide a spontaneous model of human melanoma based on an overlap between its genetic characteristics and those of some melanomas seen in people [[4], [5], [6]]. A better understanding of this disease would thus aid earlier diagnosis and treatment for canine patients and could assist in drug development for human melanomas.

MicroRNAs (miRNAs) may offer a fruitful line of research on melanomas. Aberrantly expressed miRNAs reportedly act as oncogenes or tumor suppressor genes, can alter biological functions such as cell migration, proliferation, apoptosis, and metastasis, and modulate the immune response to human melanomas [[7], [8], [9]]. miRNAs are also heavily implicated in angiogenesis for various human cancers [10]. Dysregulated miRNAs reportedly serve as potential biomarkers for stage-specific diagnosis of human melanoma and response to therapy [[11], [12], [13], [14]].

As well as acting as diagnostic biomarkers, miRNAs could meet an urgent need for therapeutic targets for COM. However, achieving therapeutic success with conventional therapies—which comprise surgical resection, radiation therapy, immunotherapy, and electrochemotherapy [15]—is regarded as challenging [16]. Researchers have focused on miRNA-based therapies because miRNAs act as potent genetic regulators; a single miRNA can modulate the cellular pathways targeting a diverse set of mRNA genes [17]. Recently, miRNA-based therapies have entered clinical trials [18]. Accordingly, the genetics and underlying molecular mechanism of COM disease progression require further elucidation for diagnostic and therapeutic advances.

Several dysregulated miRNAs have been studied in COM patients [[19], [20], [21]]. In our previous study, we identified one of these dysregulated miRNAs, miR-450b, as an oncogenic target; it was upregulated, and its target mRNA (PAX9) was downregulated in a small number of COM patients, and it was further upregulated in a metastatic (LMEC) COM cell line [19]. Aberrant miR-450b expression has been reported in human hepatic ischemia, colorectal cancer, cervical cancer, breast cancer, gastric cancer, oral squamous cell carcinoma, hepatocellular carcinoma, lung squamous cell carcinoma, and nasopharyngeal carcinoma [19,[22], [23], [24], [25], [26], [27], [28], [29], [30]]. miR-450b reportedly controls its target mRNA genes by interacting with critical signaling pathways; for example, it promotes hepatocellular carcinoma and colorectal cancer by activating PI3K/AKT and Wnt/β-Catenin signaling pathways, respectively [28,31]. However, there is a need for more information on this miRNA in COM, both in a larger number of patients and on its molecular pathways and biological functions.

Accordingly, in this study, we set out to investigate miR-450b expression in COM with the following objectives. We determined its expression level in clinical samples to evaluate miR-450b as a diagnostic biomarker for COM and metastatic progression of COM. Through *in vitro* assays with primary-tumor and metastatic melanoma cell lines (knockdown and overexpression experiments), we investigated the influence of miR-450b on cell proliferation, migration, colony formation, and apoptosis, and we further investigated its direct mRNA targets using the target scan database and its potential signaling pathways using GO and KEGG enrichment analysis.

## 2 Material and methods

### 2.1 Clinical sample information

We evaluated samples from 40 dogs in this study (age range: approx. 7–16 years; 25 males, 15 females). Oral melanoma tissue samples were obtained from thirty dogs undergoing surgery for COM at the Kagoshima University Veterinary Teaching Hospital or an affiliated local clinic between August 2009 and December 2022 and diagnosed with metastatic (n = 15) or non-metastatic (n = 15) COM. Oral tissue samples were obtained from ten dogs (healthy controls) undergoing examination at our hospital and diagnosed as tumor-free. All diagnoses were made after histopathological examination by a member of the Japanese College of Veterinary Pathologists. Plasma samples were obtained from a subset of the study population (total: n = 25; healthy controls: n = 5; non-metastatic COM: n = 10; 10 metastatic COM: n = 10). Full details of the study population are given in Table 1.

**Table 1:** COM tissue and Plasma sample information.

No	Age (Years)	Sex	Breed	WHO Stage	Metastasis Status	Tissue	Plasma
1	12.7	Male	Miniature	Ⅳ	**P**	**P**	**P**
2	14.8	Male	Mongrel	Ⅳ	**P**	**P**	**P**
3	10	Male	Golden Retriever	Ⅳ	**—**	**P**	**P**
4	10.11	Male	Miniature	Ⅰ	**—**	**P**	**—**
5	7.11	Male	Miniature	Ⅰ	**—**	**P**	**P**
6	10.9	Male	Miniature	Ⅳ	**—**	**P**	**P**
7	12	Male	Shiba	Ⅳ	**P**	**P**	**—**
8	13	Male	Pomerania	Ⅰ	**—**	**P**	**—**
9	10.3	Male	Yorkshire	Ⅳ	**—**	**P**	**P**
10	10.2	Male	Chiwawa	Ⅳ	**P**	**P**	**P**
11	12.4	Female	Miniature	Ⅳ	**—**	**P**	**P**
12	14.6	Female	Miniature	Ⅱ	**P**	**P**	**—**
13	15.2	Female	Mongrel	Ⅳ	**—**	**P**	**—**
14	12.11	Male	Miniature	Ⅳ	**P**	**P**	**—**
15	12.4	Male	Shiba	Ⅳ	**P**	**P**	**—**
16	15.2	Female	Mongrel	Ⅳ	**P**	**P**	**—**
17	10.8	Male	Miniature	Ⅳ	**—**	**P**	**—**
18	15.2	Male	Shiba	Ⅰ	**—**	**P**	**—**
19	13.3	Male	Miniature	Ⅰ	**—**	**P**	**P**
20	8.2	Female	Miniature	Ⅳ	**P**	**P**	**P**
21	12	Male	Mong	Ⅰ	**—**	**P**	**P**
22	11.1	Male	Miniature	Ⅳ	**P**	**P**	**P**
23	15.6	Male	Pomeranian	Ⅱ	**P**	**P**	**P**
24	15.3	Female	Mong	Ⅰ	**—**	**P**	**P**
25	11	Male	Miniature	Ⅳ	**P**	**P**	**P**
26	15.3	Female	Mong	Ⅰ	**—**	**P**	**P**
27	16.3	Male	Miniature	Ⅳ	**P**	**P**	**P**
28	11.8	Female	Miniature	Ⅰ	**—**	**P**	**P**
29	14	Female	Dalmatian	Ⅱ	**P**	**P**	**P**
30	12.1	Female	Toy poodle	Ⅳ	**P**	**P**	**P**

(**P**) indicates “Present,” and (−) indicates “Absent.”

Tissue samples were immersed in RNAlater immediately after collection, left at 4 °C overnight, and then stored long-term at −80 °C. Blood samples were collected in tubes treated with 3.2 % sodium citrate anticoagulant and centrifuged at 3000*g for 10 min immediately after collection. The supernatant was transferred to fresh Eppendorf tubes and centrifuged again to remove all existing cell debris at 16000*g for 10 min at 4 °C. Plasma was collected without touching the pellet and stored in a −80 °C freezer [32,33].

### 2.2 Cell lines and cell culture

In this study, we used cells from the following two COM cell lines: KMEC (primary-tumor site of origin; oral gingiva) and LMEC (metastatic site of origin; lymph node) cell lines. One co-author, Dr. Takayuki Nakagawa of Tokyo University, provided the cells. Cell culture methods and protocols accorded with the published note on these cell lines [34]. Briefly, cells were cultured using Roswell Park Memorial Institute (RPMI) media-1640 (Gibco), l-glutamine solution (Fujifilm Wako Pure Chemical Corporation, Osaka, Japan), antibiotics (penicillin-streptomycin; Sigma), and 10 % fetal bovine serum (BI, Biological Industries), and maintained at 37 °C in a controlled-humidity environment with 5 % CO_2_. The cells were then stored in liquid nitrogen with a media supplement (CultureSure®, Fujifilm Wako Pure Chemical Corporation, Osaka, Japan). Cold phosphate-buffered saline (PBS) and 0.25 % trypsin or 0.1 % EDTA were applied during the subculture. Cells were counted using an automated cell counter (LUNA II, Logos, USA).

### 2.3 miR-450b inhibitor and mimic and inhibitor and mimic negative controls

For miR-450b knockdown (1×10^5^) experiments, we used a mirVana™ miR-450b inhibitor (Cat#:4464084, Ambion; concentration: 15 nM) and miRNA Inhibitor Negative Control #1 (Cat#: 4464076, Ambion; hereafter inhibitor NC). For overexpression experiments, we used a mirVana™ miR-450b mimic (Cat#:4464066, Ambion; concentration: 10 nM) and a mirVana™ miRNA Mimic, Negative Control #1 (Cat#:4464058, Ambion; hereafter mimic NC). Transfection was performed using Lipofectamine RNAiMAX reagent (Invitrogen) and Opti-MEM media (Gibco), and cells were cultured for 48 h by the relevant assay protocol. After 48 h of transfection, a fresh medium was added.

### 2.4 RNA extraction

RNA was extracted from tissues and cells using the mirVana™ RNA Isolation Kit (Thermo Fisher Scientific) and from plasma using the mirVana™ Paris Kit (Thermo Fisher Scientific), as previously described [33,35]. Briefly, each tissue sample or the relevant cell preparation was mixed with the required amount of lysis buffer. A 300-μL aliquot of each plasma sample was mixed with an equivalent amount of 2x denaturation solution. A 1/10 amount of a miRNA homogenate additive was added to the lysate, then left on ice for 10 min. Acid-phenol: chloroform (Ambion®) was added to the tissue, cell lysate, or plasma, with subsequent thorough vortex-mixing and then centrifugation at 15000*g for 5 min at room temperature. The supernatant was then transferred carefully to an Eppendorf tube, to which a 1.25-fold amount of molecular-grade ethanol was added (and the amount recorded), and the tube contents were filtered using centrifugation. In the final step, total RNA was collected as sediment in the tube using an elution solution pre-heated to 95 °C. The total RNA level was calculated using a Nanodrop 2000c spectrophotometer (Thermo Fisher Scientific), and RNA integrity was evaluated using Bioanalyzer 2100 (Agilent). The RNA integrity number was 8.1–8.5 for the tissue and exceeded 9.0 for the cells.

### 2.5 Cell counting Kit-8 assay

KMEC and LMEC cells (2500 cells/well) were seeded onto 96-well plates and cultured for 24 h miR-450b expression was knocked down using miR-450b inhibitor at a concentration of 15 nm, and miR-450b overexpression was achieved using miR-450b mimic at a concentration of 10 nm. Inhibitor and mimic NCs were also used as comparators. The Cell Counting Kit-8 (CCk-8) reagent (Dojin Laboratories, Kumamoto, Japan) was applied, and the proliferative ability of the KMEC or LMEC cells was evaluated. In accordance with the manufacturer's protocol, 10 μL of CCK-8 reagent was applied to each well on the plates, with subsequent incubation for 2 h. Optical density was recorded at 450 nm absorbance using a MultiScan GO plate reader (Thermo Scientific) at the appropriate time intervals (0h, 24h, 48h, and 72h) post-transfection.

### 2.6 Colony formation assay

Cells (1×10^5^) were transfected with the miR-450b inhibitor or mimic, or inhibitor or mimic NC for 48 h. Transfected cells were trypsinized, counted, and seeded (2000–5000 cells/well) onto six-well plates and cultured for 8–10 days at 37 °C. A typical colony was considered to be at least 50 cells. In accordance with a published protocol, cells were washed twice with cold PBS. A mixture of 2–3 ml of 6.0 % glutaraldehyde and 0.5 % crystal violet was added, and the plates were left for 30 min. The mixture was then washed off by careful rinsing with water. The colony plates were left at 20 °C for drying [36]. The colony was observed under the stereomicroscope and photographed using a digital camera. Image J analysis software was used to count the colonies. The experiment was conducted in triplicate.

### 2.7 Monolayer wound healing assay

A wound-healing assay was performed to measure the effects of miR-450b inhibition and overexpression. Briefly, cells (1×10^5^) were seeded onto 24-well plates and transfected with the miR-450b inhibitor or mimic, or inhibitor or mimic NC, and cultured for 48 h. The medium was removed and washed twice with cold PBS. A scratch line was carefully drawn on the cell monolayer from a forward to a backward position using sterilized pipette tips (200 μL). Cell debris was washed out using PBS, and the preparation in the medium was suspended. Wound healing time was recorded at the appropriate length of time. The wound's width was measured using ToupView (https://sios.net.au/software/toupview) software. The experiment was conducted in triplicate.

### 2.8 Transwell migration assay

Cells were seeded onto 24-well plates, transfected with miR-450b inhibitor or mimic, or inhibitor or mimic NC, and cultured for 48 h. The cells were then trypsinized, counted, and suspended (5×10^4^) in 300 μL of serum-free DMEM medium into the upper compartment of the relevant transwell insert (6.5 mm insert, 8 μm pore, 24 well insert, Costar). 700 μl of DMEM (Dulbecco's Modified Eagle Medium) medium containing 10 % fetal bovine serum was added to the wells on a 24-well plate, and the plate was incubated at 37 °C for 24 h. The migrated cells were then washed with ice-cold PBS. The cells were fixed for 2 min in 4 % formaldehyde and permeabilized for 20 min in 100 % methanol at room temperature. Cells were stained with a 0.5 % crystal violet solution and left for 15 min. Non-migrated cells were removed with a sterile dry swab. The transwell insert was observed under the microscope, and images were randomly captured. Image J software was used to count the cells in each field. The experiment was conducted in triplicate.

### 2.9 TUNEL Alexa Fluor imaging assay

Apoptotic effects on cells were investigated in accordance with the manufacturer's protocol (Invitrogen). In brief, cells (5000 cells/well) were transfected with the miR-450b inhibitor or mimic, or inhibitor or mimic NC, on a 96-well plate for 48 h. After removing the medium and washing with PBS, the cells were fixed (in 4 % paraformaldehyde for 15 min) and permeabilized (in 0.25 % Triton X-100 for 20 min). TdT reaction buffer was added, and the cells were left to stand for 10 min. TdT reaction cocktail was then added, and the plate was left to stand for 60 min at 37 °C. Click-iT reaction cocktail was then added, and the plate was left to stand for 30 min. After removing the Click-iT reaction cocktail, the DNA nuclei were stained with Hoechst 33342 antibody. Imaging was carried out using a KEYENCE fluorescence microscope (BZ-X series). The experiment was conducted in triplicate.

### 2.10 Flow cytometry using Annexin V-Biotin/PI staining

A flow cytometer (BD Biosciences) was used to detect the percentage of apoptotic cells versus total cells, following transfection with the miR-450b inhibitor or mimic, or inhibitor or mimic NC for 48 h, using an Annexin V-Biotin and Propidium iodide (PI) Kit, in accordance with the manufacturer's instructions (Bio Vision). In short, cells (1×10^5^) were suspended in 200 μL of 1X binding buffer. Annexin V-Biotin and PI were added to the suspension, which was left to stand in the dark for 5 min. The cells were centrifuged at 2300*g for 2 min to remove the binding buffer. Following washing with 200 μL of 1X binding buffer, the cells were fixed in 2 % formaldehyde for 15 min, stained with avidin-fluorescein, and kept at room temperature for 15 min. Finally, cells were analyzed for apoptosis using a FACSCalibur flow cytometer (BD Biosciences, Franklin Lakes, NJ, USA), and data were analyzed using FlowJo v10 (Tree Star) software. Annexin V (+)/PI (−) indicated early apoptosis. The experiment was conducted in triplicate.

### 2.11 Quantitative real-time PCR (qRT-PCR)

To assess the relative expression of miR-450b (TaqMan ID: 006407) in tissues, plasma, and cell lines, a qRT-PCR was performed as previously described [33,35]. First, 1.25 μL (2 ng/μL for tissues or cells) of total RNA was reverse transcribed to cDNA in a T100 thermal cycler (Bio-Rad) using the TaqMan MicroRNA Reverse Transcription Kit (Thermo Fisher Scientific) in accordance with the manufacturer's protocol. Plasma was spiked with miR-cel-39 to ensure the same amount of RNA isolation. A TaqMan First Advanced Master Mix Kit and a Quant Studio 3 real-time PCR system (Thermo Fisher Scientific) were applied for qRT PCR. Expression values were normalized using internal controls, RNU6B for tissues and cells, or miR-16 for plasma samples. To make cDNA, 250 ng of total RNA was reverse transcribed for the target mRNA genes using ReverTra Ace qPCR RT master mix with gDNA Remover (Toyobo, Japan). The qRT-PCR procedure was the same as explained above. GAPDH was used as an internal control for normalizing the mRNA expression level. TaqMan gene assay targets were GAPDH (ID: Cf04419463_gH), PAX9 (ID: Cf02705737_m1), MMP9 (ID: Cf02621845_m1), and BMP4 (ID: Cf01041266). Expression was quantified using the 2^−ΔΔCT^ method. The acceptable Ct value was less than or equal to 36.

### 2.12 Pathway and gene ontology analyses

To investigate the pathways involved with miR-450b Kyoto Encyclopedia Genes and Genomes (KEGG) pathway and Gene Ontology (GO) analyses were performed. A p-value of <0.05 was considered as the threshold to define statistical significance.

### 2.13 Statistical analysis

All statistical analyses were conducted using GraphPad Prism 9 (GraphPad Software, Inc., USA). One-way ANOVA was applied to determine the relative expression value, followed by the Kruskal-Wallis and Mann-Whitney U tests. Two-way ANOVA was applied to the results of time-dependent experiments, followed by Sidaq's multiple comparisons. ROC curves were constructed to assess the performance of miR-450b as a model for predicting metastatic or non-metastatic COM using Wilson and Brown's method. P values < 0.05 were considered significant.

## 3 Results

### 3.1 Relative expression of miR-450b in clinical samples indicates its promise as a biomarker

To evaluate miR-450b as a potential biomarker, we investigated its expression level in clinical tissues using qRT-PCR. For oral tissue samples, miR-450b was significantly upregulated in metastatic COM (Fold change; FC = 495, P = 0.0001) and non-metastatic COM (FC = 45, P = 0.0001) versus healthy controls (Fig. 1 A). For plasma samples, miR-450b was preferentially upregulated in metastatic COM (FC = 9, P = 0.0001) and non-metastatic COM (FC = 9, P = 0.0001) versus healthy controls (Fig. 1B). Our data also showed that miR-450b expression—in both oral tissue (P = 0.0001) and plasma (P = 0.004)—predicted whether the COM was metastatic or not. ROC analysis revealed areas under the curve (with p-values) of 0.93 (0.0037) for healthy controls vs. COM overall, 0.86 (0.027) for healthy controls vs. non-metastatic COM, 1.0 (0.002) for healthy controls vs. metastatic COM, and 0.88 (0.005) for non-metastatic versus metastatic COM (Fig. 1C–F), further confirming that miR-450b is a potential biomarker for COM and that its level of upregulation can differentiate between metastatic and non-metastatic cases.

**Fig. 1:**
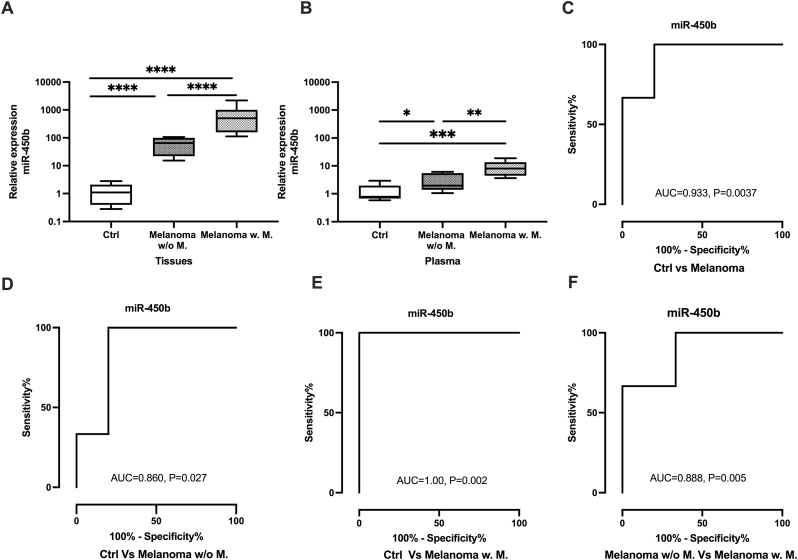
**Relative expression of miR-450b in clinical samples.** (A). Relative expression of miR-450b in healthy oral tissues (control, n = 10) and canine oral melanoma tissues (Metastatic, n = 15, no metastatic, n = 15). (B). Expression of miR-450b in plasma samples. (C–F). ROC curve analysis of miR-450b to measure the potentiality as the biomarker. One-way ANOVA followed by Tukey's multiple comparisons and Mann-Whitney *U* test were used for statistical analysis. The Y-axis represents the relative expression level of miR-450b in log10 units. *P < 0.05, **P < 0.01, ***P < 0.001, ****P,0.0001, ctrl; control, w/o M.; without metastasis, w. M.; with metastasis.

### 3.2 miR-450b in COM cell lines: relative expression, knockdown, and overexpression

To further confirm the potential of miR-450b to identify metastasis, we investigated its expression in KMEC and LMEC cell lines (Primary-tumor and metastatic origins, respectively) using qRT-PCR. mir-450b expression level was significantly higher in LMEC (FC = 8, P = 0.007) than KMEC cells (Supplementary Fig. 1 A). We then evaluated its expression in these cell lines after knockdown and overexpression (application of miR-450b inhibitor at 10 nM, 15 nM, and 20 nM or mimic at 10 nM and relevant NC). For both KMEC (P = 0.002) and LMEC (P = 0.002) cells, relative miR-450b expression was significantly reduced with knockdown (inhibitor at 15 nm; Supplementary Fig. 1 B, C) and significantly elevated with overexpression (mimic at 10 nm; Supplementary Fig. 1 D, E), providing a further demonstration that miR-450b is upregulated in COM cell lines, with greater upregulation in the cell line of metastatic origin.

### 3.3 miR-450b promotes cell proliferation and clonogenicity

To assess the influence of miR-450b on proliferation and clonogenicity, CCK-8 and clonogenic assays were performed in KMEC and LMEC cells. The CCK-8 assay revealed significantly decreased cell proliferation at 48h post-transfection in LMEC (P = 0.01) and KMEC (P = 0.004) cells, with miR-450b knockdown (Fig. 2 A, B) and significantly increased cell proliferation at 48h or 72h post-transfection in LMEC (P = 0.01) and KMEC (P = 0.0001) cells, with miR-450b overexpression (Fig. 2 C, D).

**Fig. 2:**
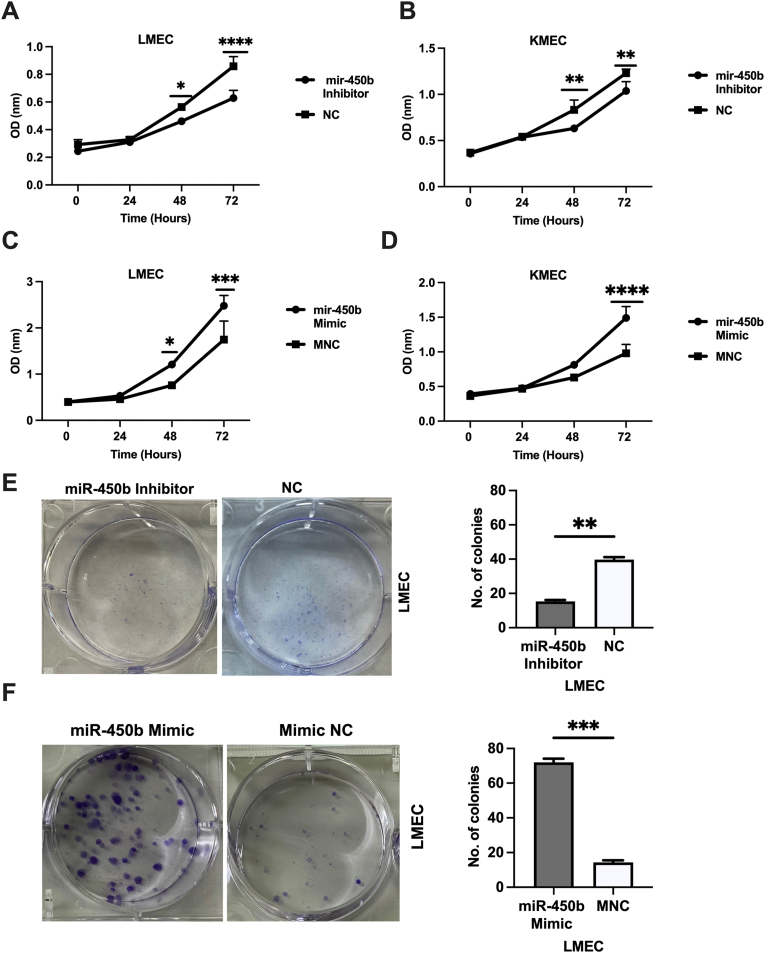
Effects of miR450b on cell proliferation and colony formation in canine oral melanoma cell lines. (A, B). CCK8 assay of miR-450b inhibitor (C, D). miR-450b mimic was carried out in LMEC and KMEC cell lines in a time-dependent manner. (E). Colony formation assay of miR-450b inhibitor and (F). miR-450b mimic performed in LMEC cell line. Cells>50 in number were scored. The number of colonies was measured by Image J software. The data represents the colony count ± SEM (right). Results are representative of three independent experiments. Two-way ANOVA followed by Sidaq's multiple comparisons was used for the CCK8 assay. *P < 0.05, **P < 0.01, ***P < 0.001, ****P,0.0001.

The clonogenic assay revealed significantly reduced colony numbers (average: 15 vs. 39 for NC; P = 0.005) with miR-450b knockdown and significantly elevated colony numbers (average: 72 vs. 14 for NC; P = 0.0002) with miR-450b overexpression, in LMEC cells (Fig. 2 E, F). However, no colony was observed in KMEC cells after knockdown or overexpression (Suppl. Fig. 2). Thus, we consider that miR-450b may be involved in the metastatic progression of COM rather than its primary stage. Taking our findings together, miR-450b appears to promote cell proliferation in both cell lines and to promote clonogenicity in metastatic COM (LMEC cell line).

### 3.4 miR-450b influences cell migration

To investigate the effects of miR-450b inhibition and overexpression in LMEC and KMEC cells, we performed wound-healing and transwell migration assays.

In the wound-healing assay, miR-450b knockdown significantly inhibited cell migration; the scratch area was non-overlapped at 24h post-transfection in LMEC (mean width = 0.35 mm, P < 0.0001) and KMEC (mean width = 0.19 mm, P = 0.04) cells (Fig. 3 A, B). Conversely, miR-450b overexpression significantly increased cell migration; the scratch area was overlapped at 18 h and 24 h (P < 0.0001) in KMEC cells (vs. NC widths of o.22 mm and 0.26 mm) (Fig. 3 C, D).

**Fig. 3:**
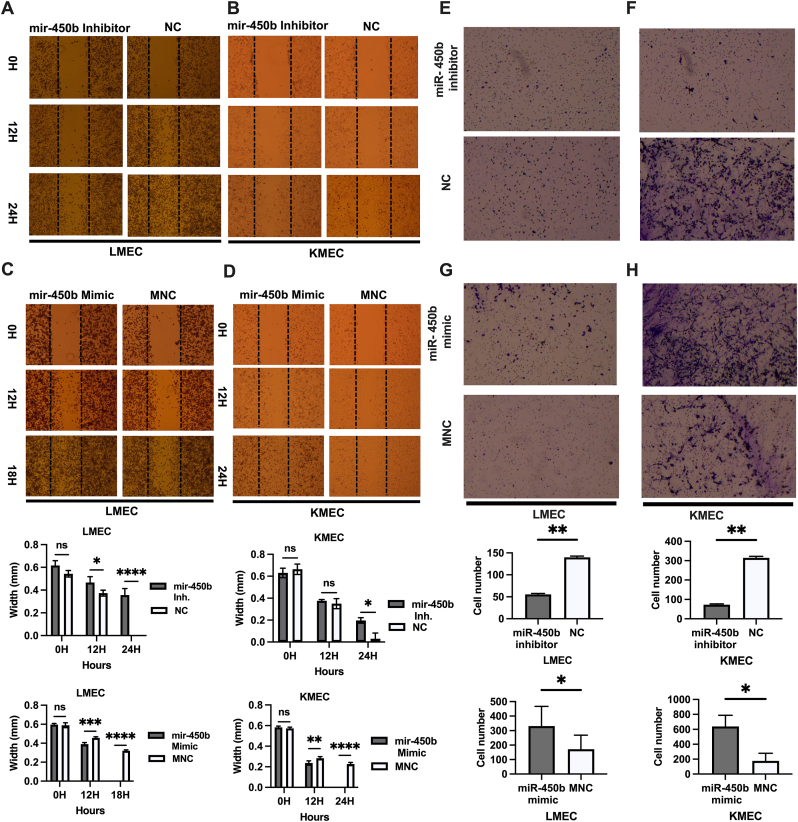
**Effects of miR-450b knockdown and overexpression on Canine oral melanoma cell migration.** (A–D). The effect of miR-450b inhibition and miR-450b mimic on cell migration in LMEC and KMEC cell lines was analyzed using the wound healing assay. Representative images of the wound healing (Upper left) and calculated scratch area (Upper right) were illustrated. (E–H). Transwell migration assay with miR-450b inhibitor and miR-450b mimic in LMEC and KMEC cells (lower panel). The number of migrated cells was measured by Image J software. Results are representative of three independent experiments. The data represents the cell count ± SEM (lower right). Two-way ANOVA followed by Sidaq's multiple comparisons for wound healing assay. *P < 0.05, **P < 0.01, ***P < 0.001, ****P,0.0001, ns; not significant.

In the transwell migration assay, miR-450b knockdown yielded smaller numbers of LMEC (average of 55 cells migrated from the upper chamber of the transwell insert vs. average 140 with NC; P = 0.003) and KMEC (average 73 cells vs. 314 with NC; P = 0.006) cells (Fig. 3. E, F). Conversely, miR-450b overexpression significantly increased migration ability in LMEC (average 331 vs. 171 cells, P = 0.02) and KMEC (average 638 vs. 176 cells, P = 0.04) cells (Fig. 3 G, H). In summary, miR-450b knockdown decreased, and overexpression increased cell migration.

### 3.5 miR-450b inhibits apoptosis

**Fig. 4:**
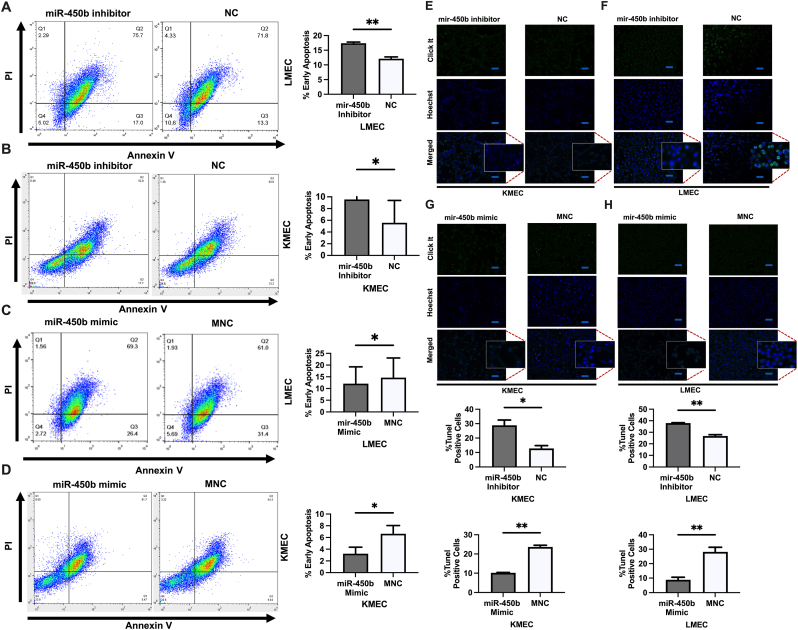
**Effects of miR-450b knockdown and overexpression on cell Apoptosis**. (A–D). Annexin V-Biotin/PI, staining, and flow cytometry showed the percentages of early apoptosis in LMEC and KMEC cell lines treated with miR-450b inhibitor and miR-450b mimic, respectively. (E–H). Tunel assay illustrated the percentage of Tunel-positive cells in LMEC and KMEC cell lines by miR-450b inhibitor and miR-450b mimic, respectively. The squared box indicates TUNEL-positive (Cyan-blue colored) and TUNEL-negative cells (didn't produce cyan-blue color), and the images were captured with 10X magnification power (scale bar = 50 μm). Results are representative of three independent experiments. The data represents the cell count ± SEM. *P < 0.05, **P < 0.01.

To further elucidate the molecular mechanism underlying the functions of miR-450b, we performed a flow cytometry assay and TUNEL Alexa Fluor imaging assay investigating the effects of miR-450b on apoptosis. Flow cytometry revealed significant percentages of cells experiencing early apoptosis with miR-450b knockdown for both the LMEC (17.4 % vs. 12.1 % for NC) and KMEC (9.56 % vs. 5.28 %) cell lines (Fig. 4 A, B). This pattern was completely reversed with overexpression: the percentages of cells experiencing early apoptosis were reduced for the LMEC (12.0 % vs. 14.64 %) and KMEC (3.46 % vs. 6.63 %) cell lines (Fig. 4 C, D). Although the percentages of early apoptosis were statistically significant, the apoptosis rates difference between knockdown or overexpression and their subsequent NCs were minimal. So, we posit that miR-450b has minimum inhibitory effects on cell apoptosis.

The TUNEL Alexa Fluor imaging assay revealed greater percentages of TUNNEL-positive cells after miR-450b knockdown for both the LMEC (38 % vs. 27 % for NC) and KMEC (29 % vs. 13 %) cell lines (Fig. 4E and F). This trend in percentages was reversed with overexpression: the percentages of TUNNEL-positive cells were reduced for both the LMEC (9 % vs. 29 %) and KMEC (10 % vs. 23 %) cell lines (Fig. 4 G, H).

Thus, our flow cytometry findings were further substantiated by the TUNEL Alexa Fluor imaging assay, and considered together, our findings suggest that miR-450b inhibits apoptosis.

### 3.6 Targets of miR-450b and the probable predictive pathways

To determine the probable miR-450b-related pathway, we performed GO enrichment and KEGG pathway analyses. We confirmed that PAX9 was the potential binding target of miR-450b (Supplementary Fig. 3 A). In addition, our previous study investigated that miR-450b correlated with BMP4 and MMP9 based on NGS results [19].

All genes related to miR-450b were curated using a target scan database (https://www.targetscan.org/vert_80/) and submitted to the DAVID bioinformatics database (https://david.ncifcrf.gov/), revealing that miR-450b is associated with cell proliferation, differentiation, aging, and response to hypoxia, and may follow the cAMP/calcium signaling/FoxO signaling pathways. KEGG and GO enrichment analysis suggests miR-450b is involved in the Wnt signaling pathway (Supplementary Fig. 3 B, C).

### 3.7 Relative expression of PAX9, BMP4, and MMP9 in COM and cell lines

**Fig. 5:**
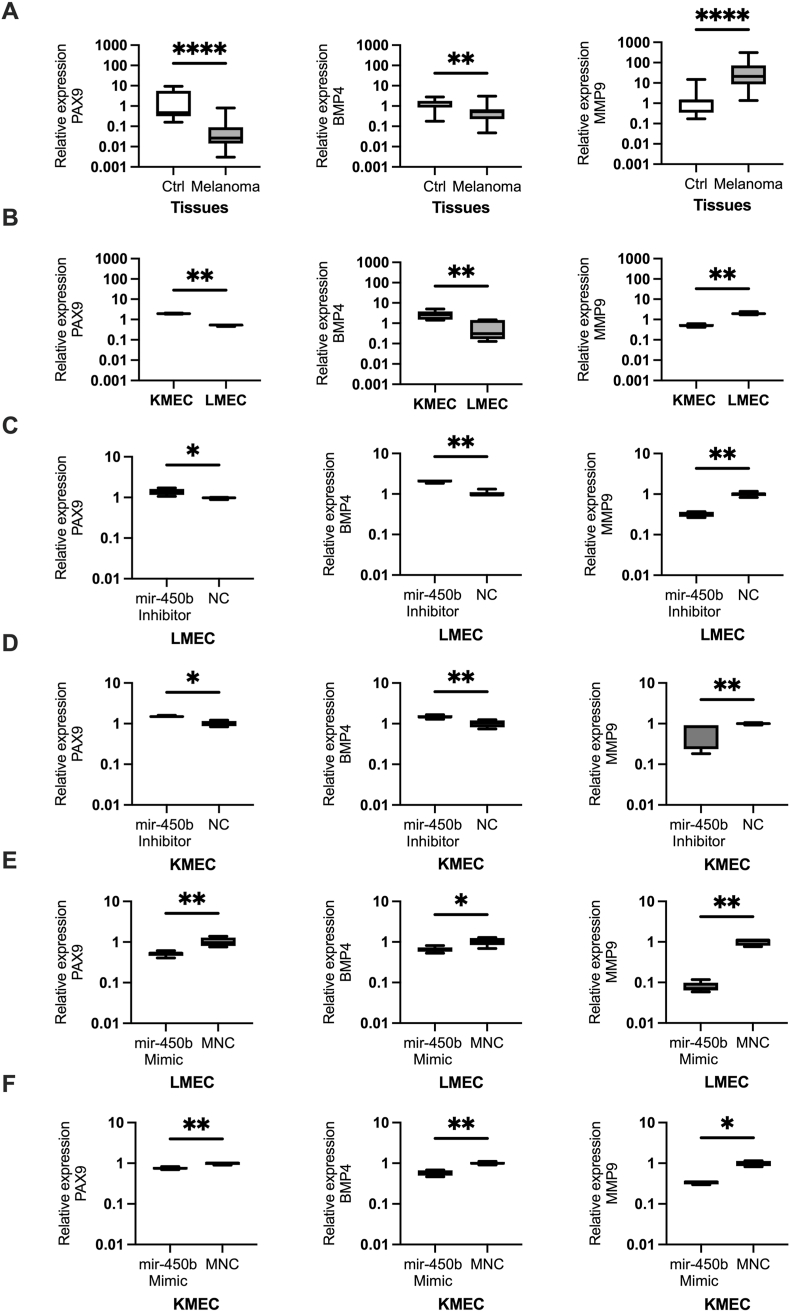
The expression levels of PAX9, BMP4, and MMP9 mRNA in clinical samples and cell lines. (A). Relative expression of PAX9, BMP4, MMP9 in canine oral melanoma tissue samples (Control, n = 10, Melanoma, n = 30) and (B). KMEC and LMEC cell lines. (C, D). The expression level of PAX9, BMP4, and MMP9 in indicated cells after the knockdown of miR-450b**.** (E, F). The expression level of PAX9, BMP4, and MMP9 in indicated cells after the overexpression of miR-450b. Student t-test followed by the Mann-Whitney *U* test was used for statistical analysis. The Y-axis represents the relative expression level of mRNAs in log10 units. *P < 0.05, **P < 0.01, ****P,0.0001.

To investigate miR-450 b's target mRNA axis, we investigated the expression of PAX9, BMP4, and MMP9 using qRT-PCR in our clinical tissue samples (n = 30) and in the KMEC and LMEC cell lines. In COM tissues, PAX9 (FC = 0.04, P < 0.0001) and BMP4(FC = 0.57, P = 0.003) were significantly downregulated, whereas MMP9 (FC = 24.6, P < 0.0001) was significantly upregulated (Fig. 5. A).

In the cell lines, PAX9 (P = 0.002) and BMP4 (P = 0.008) showed elevated expression in KMEC versus LMEC (FC = 3.80 vs. 0.26, and FC = 4.32 vs. 0.23, respectively) cells. MMP9 (P = 0.002) expression was reduced in KMEC (FC = 0.26) versus LMEC (FC = 0.26 vs. 3.81) cells (Fig. 5 B). Our findings suggest that PAX9 and BMP4 are downregulated, MMP9 is upregulated in COM, and these expression patterns may be more pronounced in metastatic COM.

### 3.8 miR-450b knockdown and overexpression altered the expression of PAX9, BMP4, and MMP9

To verify whether the expression pattern of the PAX9, BMP4, and MMP9 mRNA transcript level in COM is directly affected by miR-450b, we evaluated the expression of these target mRNAs in LMEC and KMEC cell lines after miR-450knockdown and overexpression with qRT-PCR analysis. Knockdown of miR-450b significantly elevated PAX9 (respective LMEC and KMEC cells: P = 0.03, P = 0.02) and BMP4 (P = 0.004, P = 0.002), but significantly decreased MMP9 (P = 0.004, P = 0.009) expression levels (Fig. 5 C, D). Contrastingly, miR-450b overexpression significantly decreased PAX9 (P = 0.002, P = 0.009), BMP4 (P = 0.01, P = 0.009), and MMP9 (P = 0.004, P = 0.01) in LMEC and KMEC cells (Fig. 5 E, F). Although overexpression did not yield the expected change in MMP9 expression, our overall results demonstrate that miR-450b directly influences PAX9, concomitantly affecting BMP4 and MMP9 expression in COM.

## 4 Discussion

Here, we present the first evidence on the functional role of miR-450b in veterinary oncology, specifically in COM. We also furnish evidence on the utility of miR-450b as a biomarker for the disease. In the first stage of our study, we demonstrated that miR-450b is a potential marker of COM and can distinguish metastasized from non-metastasized COM. miR-450b was upregulated in COM, in both plasma and tumor tissue, clinically. This study thus substantiates our previous report on upregulated miR-450b (Rahman et al., 2019), and the current study represented a comprehensive evaluation with a larger study population (n = 30 vs n = 17) with both tumor tissue and plasma samples. As another novel feature of this study, the results from COM clinical samples were subjected to ROC analysis, which revealed that miR-450b is an indicator of COM, even at the non-metastatic stage. We thus consider our evidence to make a compelling case for miR-450b as a potential biomarker for COM.

Interestingly, our evaluation of clinical (tumor tissue and plasma) sample revealed that miR-450b could also act as a marker of COM metastasis, as it is apparently able to differentiate between metastatic and non-metastatic COM. We further investigated this potential maker utility in primary-tumor and metastatic-origin COM cell lines (KMeC and LMeC, respectively) and found miR-450b was preferentially elevated in the metastatic-origin cell line (LMeC). Our findings raise the possibility that miR-450b is implicated in progression to metastasis in COM. This possibility is consistent with recent reports implicating elevated miR-450b expression in disease progression for human oral squamous cell carcinoma, colorectal cancer, lung cancer, and esophageal squamous cell carcinoma [22,31,37,38].

At the next stage of our study, we evaluated the biological functions of mir-450b in COM through a series of assay (migration assay, wound-healing assay, colony formation assay, Alexa Fluor imaging and Flow cytometry in KMeC and LMeC cells subject to miR-450b knockdown and overexpression). Taken together, our findings in these assays indicate that miR-450b promotes cell proliferation, migration, and clonogenicity and inhibits in COM, based on the consistent pattern in which miR-450b knockdown inhibited cell proliferation, migration, and colony formation and promoted apoptosis, and the exact opposite pattern yielded by miR-450b overexpression. However, miR-450b knockdown and overexpression did not produce any colony in KMeC cells. We postulated that miR-450b is involved in metastatic progression in COM. This is consistent with reports on miR-450b modulation of cell proliferation, migration, invasion, colony formation, apoptosis, and metastasis in human hepatic ischemia, colorectal cancer, cervical cancer, breast cancer, gastric cancer, oral squamous cell carcinoma, hepatocellular carcinoma, lung squamous cell carcinoma, and nasopharyngeal carcinoma [[22], [23], [24], [25], [26], [27], [28], [29], [30]].

In this study, we also further investigated the effects of miR-450b on target mRNA genes in COM, specifically PAX9, BMP4, and MMP9. We set out by replacing the pattern reported in our previous study, where we first postulated that miR-450b has effects on these target genes may be implicated in the metastatic progression of COM [19]. Specifically, the relevant pattern involved downregulated PAX9 and BMP4 and upregulated MMP9 with upregulated miR-450b. Our suggestion that miR-450b may promote metastatic COM through its effects on these target genes appears plausible, considering that miRNAs can alter cancer growth and progression by targeting the 3′ untranslated region (UTR) of different mRNA genes and can control multiple signaling pathways involved in cancer growth [39]. We next demonstrated that miR-450b exerts a direct effect on the PAX9, BMP4, and MMP9 through knockdown and overexpression assays in LMEC and KMEC cell lines (miR-450b knockdown yielded upregulated PAX9 and BMP4 and downregulated MMP9, whereas overexpression yielded downregulated PAX9 and BMP4). In our previous study, we hypothesized this expression pattern and its link to metastasis, considering that MMP9 may correlate with tumor metastasis as it induces degradation of the extracellular matrix, which is a prerequisite for tumor invasion, and our findings here provide further support for that hypothesis.

We suggest that miR-450b regulates PAX9 functions and that PAX9 interacts with BMP4 downregulation, concomitantly affecting MMP9 expression, and this phenomenon is consistent with what is known about the target miRNAs. PAX9 is involved in early tumor development, modulates cellular function, and may induce carcinogenesis [40,41]. Its biological functions are little studied in the field of either human or canine oncology. PAX9 is reportedly involved in cell proliferation, migration, and apoptosis in oral squamous cell carcinoma, cervical cancer, and oro-esophageal epithelial cancer [[42], [43], [44]], and is an essential transcription factor in tooth development and palate morphogenesis that can modulate the expression of BMP4 expression [45,46]. BMP4 is reportedly involved in human malignant melanoma and alters biological functions [[47], [48], [49]], and inhibits MMP9 expression in cancer cells [50,51]. MMP9 is involved in the melanogenesis pathway and is considered a promising biomarker and therapeutic target for managing melanoma patients [[52], [53], [54]].

Our study also has some implications for using COM as a spontaneous disease model of human melanoma. The sequence of miR-450b differs by only one nucleotide between humans and dogs, although the human and canine seed sequences are identical in length. To the best of our knowledge, miR-450b has not been studied in human melanoma, but discovering whether it has the same diagnostic and therapeutic potential in human and canine medicine would appear to be a fruitful line of research.

In general, miR-450b regulates its target mRNA genes by activating critical signaling pathways [26,28,31,55]. Our study further explored the probable predictive pathways of miR-450b in COM progression. KEGG pathway analysis revealed that miR-450b might be involved in cAMP/calcium signaling/FoxO signaling pathways. However, our GO enrichment and KEGG pathway analysis results provide further evidence that miR-450b would be involved in the Wnt signaling pathway. miR-450b reportedly directly binds with the 3′-UTRs of SFRP2 and SIAH1 and activates Wnt/β-Catenin signaling pathways [31]. Multiple studies have revealed that melanoma progression occurs by activating the Wnt singling pathway [[56], [57], [58], [59], [60], [61], [62]]. Taken together with these previous reports, our results suggest that the Wnt signal is a crucial pathway for melanoma development involving the target genes of miR-450b.

The present study has some limitations. We did not evaluate miR-450b *in vivo* (for example, in a mouse model) or measure the protein expression of mRNA genes. Furthermore, we investigated only three genes (target mRNAs) here; however, it is likely that other mRNAs are targeted by miR-450b directly or indirectly.

Altogether, we propose a model where miR-450b upregulation inversely regulates PAX9 functional expression, and there is an interplay between the degradation of PAX9 function and BMP4 downregulation, resulting in MMP9 upregulation, through probable activation of the Wnt signaling pathway in COM (Supplementary Fig. 4). miR-450b exerts its function by promoting cell proliferation, migration, and clonogenicity and inhibiting apoptosis.

## 5 Conclusions

Based on this comprehensive evaluation of miR-450b expression, and its biological function and molecular mechanisms, we anticipate that miR-450b possesses utility as a diagnostic biomarker for COM and for distinguishing between metastatic and non-metastatic COM. We conclude that miR-450b may promote cell proliferation, migration, and colony formation, inhibit apoptosis, and trigger BMP4 downregulation and subsequent MMP9 upregulation by directly binding the 3′ UTR of PAX9 and modulating that target mRNA's function. We further suggest that miR-450b regulates target genes by activating the Wnt singling pathway. We believe that miR-450b could be a therapeutic target for COM and even human melanomas, as well as a potential biomarker.

## 6 Informed consent statement

This study was approved by the ethics committee of the Kagoshima University Veterinary Teaching Hospital (Approval No. KVH220001) and was conducted in accordance with the regulations of this committee and Kagoshima University. All samples from dogs were obtained with the consent of the relevant owner.

## Funding

This work was supported by 10.13039/501100001691JSPS KAKENHI (Grant nos. 21H02366, 20K21375) and Japan-Germany Research Cooperative Program between 10.13039/501100001691JSPS and 10.13039/100021828DAAD, grant number JPJSBP120223507.

## Declaration of Competing interest

The authors declare that they have no conflict of interest.

## CRediT authorship contribution statement

**MD Nazmul Hasan:** Writing – original draft, Visualization, Validation, Methodology, Investigation, Formal analysis, Conceptualization. **Md. Mahfuzur Rahman:** Writing – original draft, Visualization, Validation, Investigation, Formal analysis, Conceptualization. **Al Asmaul Husna:** Investigation, Formal analysis. **Mohammad Arif:** Visualization, Validation. **Indre Jasineviciute:** Visualization, Validation, Investigation, Formal analysis. **Daiki Kato:** Visualization, Validation, Investigation, Formal analysis. **Takayuki Nakagawa:** Visualization, Validation, Investigation, Formal analysis. **Naoki Miura:** Writing – review & editing, Resources, Project administration, Methodology, Funding acquisition, Conceptualization.

## Figures and Tables

**figs1:**
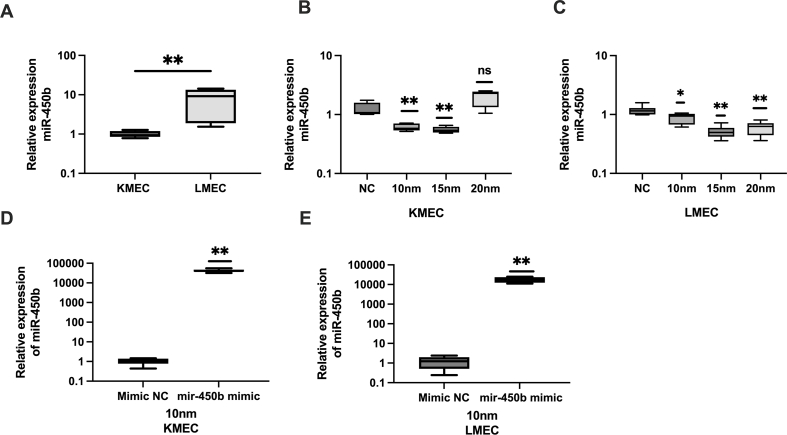


**figs2:**
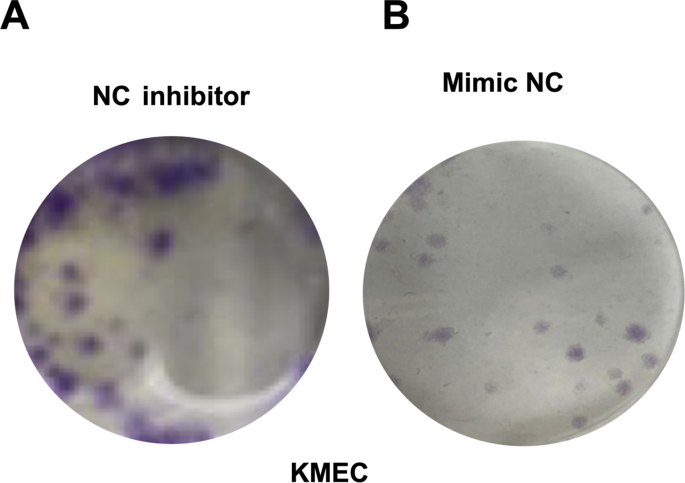


**figs3:**
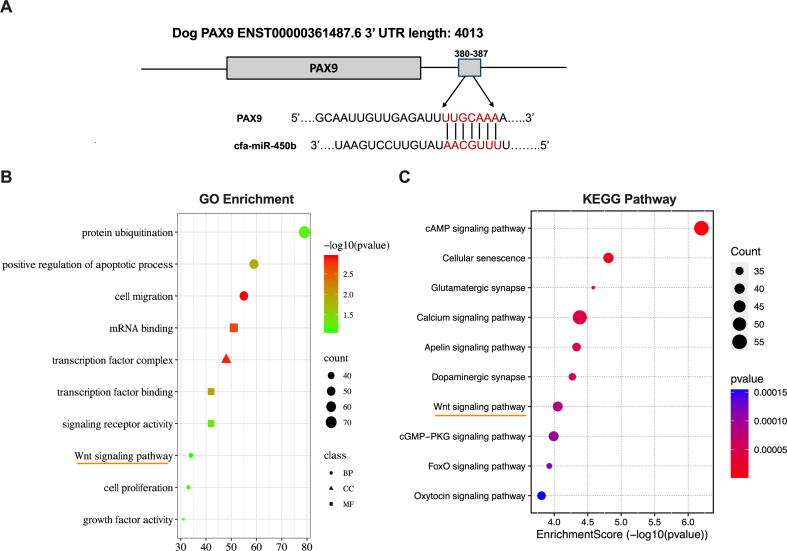


**figs4:**