# Supplementary material for: Upregulation and functional roles of miR-450b in canine oral melanoma
Source: Noncoding RNA Res. 2024 Feb 1;9(2):376–87. doi: 10.1016/j.ncrna.2024.01.017 (PMC10950611; doi:10.1016/j.ncrna.2024.01.017)
Supplement: Multimedia component 1 [file mmc1.docx]

**Supplementary Fig. 1. Relative expression of miR-450b inhibitor and miR-450b mimic in KMEC and LMEC cell lines.** (A). Relative of expression of miR-450b in KMEC and LMEC cell lines. (B, C). Relative expression of miR-450b after transfected with miR-450b inhibitor for 48 h in KMEC and LMEC cell lines, respectively. (D, E). Relative expression of miR-450b after transfection with miR-450b mimics for 48 h in LMEC and KMEC cell lines, respectively. One-way ANOVA followed by Tukey’s multiple comparisons and Mann-Whitney U test were used for statistical analysis. The Y-axis represents the relative expression level of miR-450b in log10 units. *P<0.05, **P<0.01, ns; not significant, NC; negative control.

**Supplementary Fig. 2**. **Colony formation** of (A) KMEC NC inhibitor and (B) NC mimic cells. The number of colonies was measured using Image J software. The data represents the colony count ± SEM (right). Results are representative of three independent experiments.

**Supplementary Fig. 3. Identification of miR-450b target genes in canine oral melanoma and their potential pathways.** (A). The schematic diagram illustrated the binding sites of miR-450b to PAX9 mRNA. (B, C). GO enrichment and KEGG enrichment of pathways involving predicted miR-450b targeting genes.

**Supplementary Fig. 4. An illustration of the proposed model for miR-450b and its target mRNA.** Upregulation of miR-450b inversely regulates the PAX9 functions, and degradation of PAX9 function could interplay with BMP4 downregulation, resulting in MMP9 upregulation in COM by activating Wnt signaling pathways. miR-450b exerts its function by promoting cell proliferation, migration, clonogenicity, and inhibiting cell apoptosis.

**
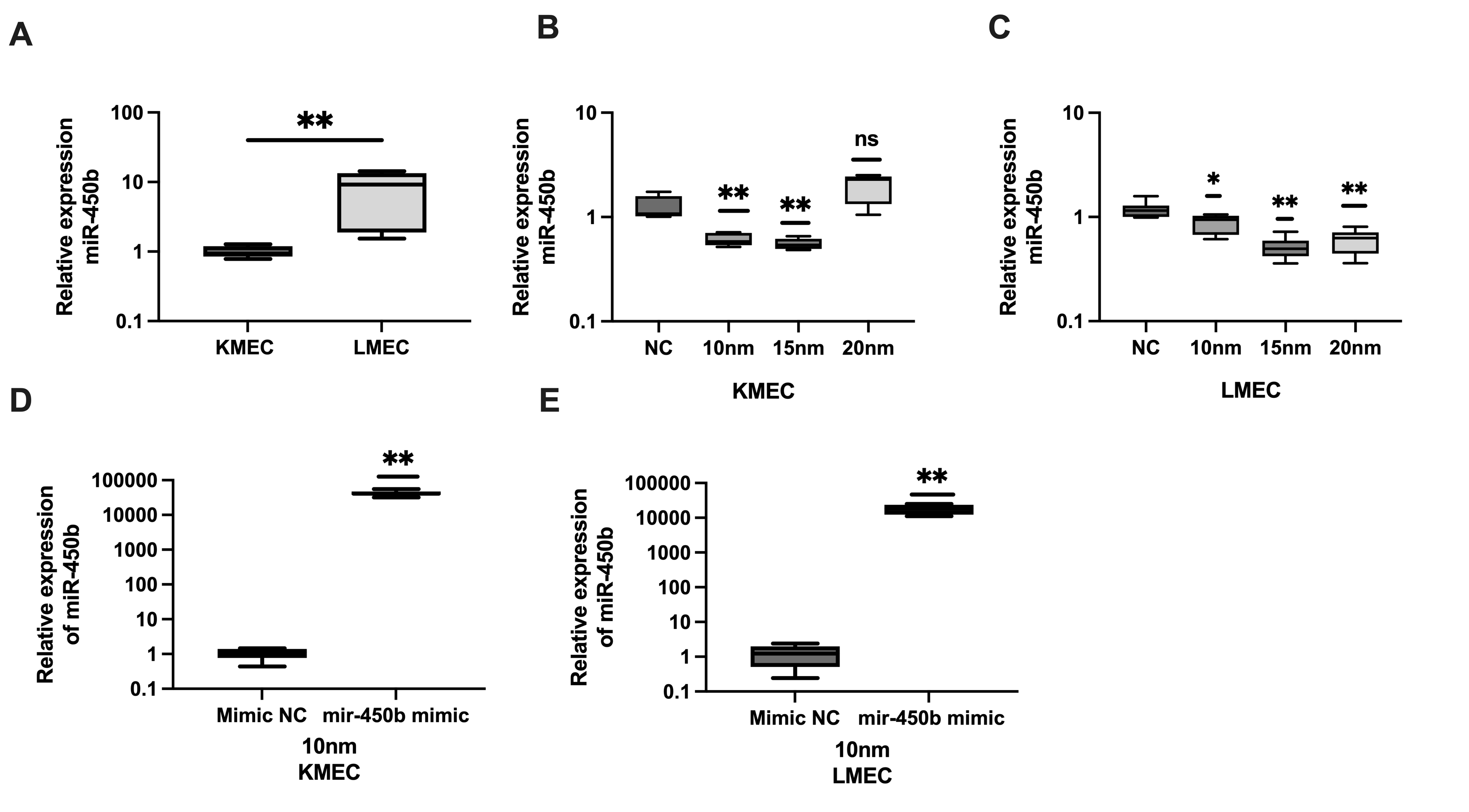
Figures**

**Supplementary Fig. 1**


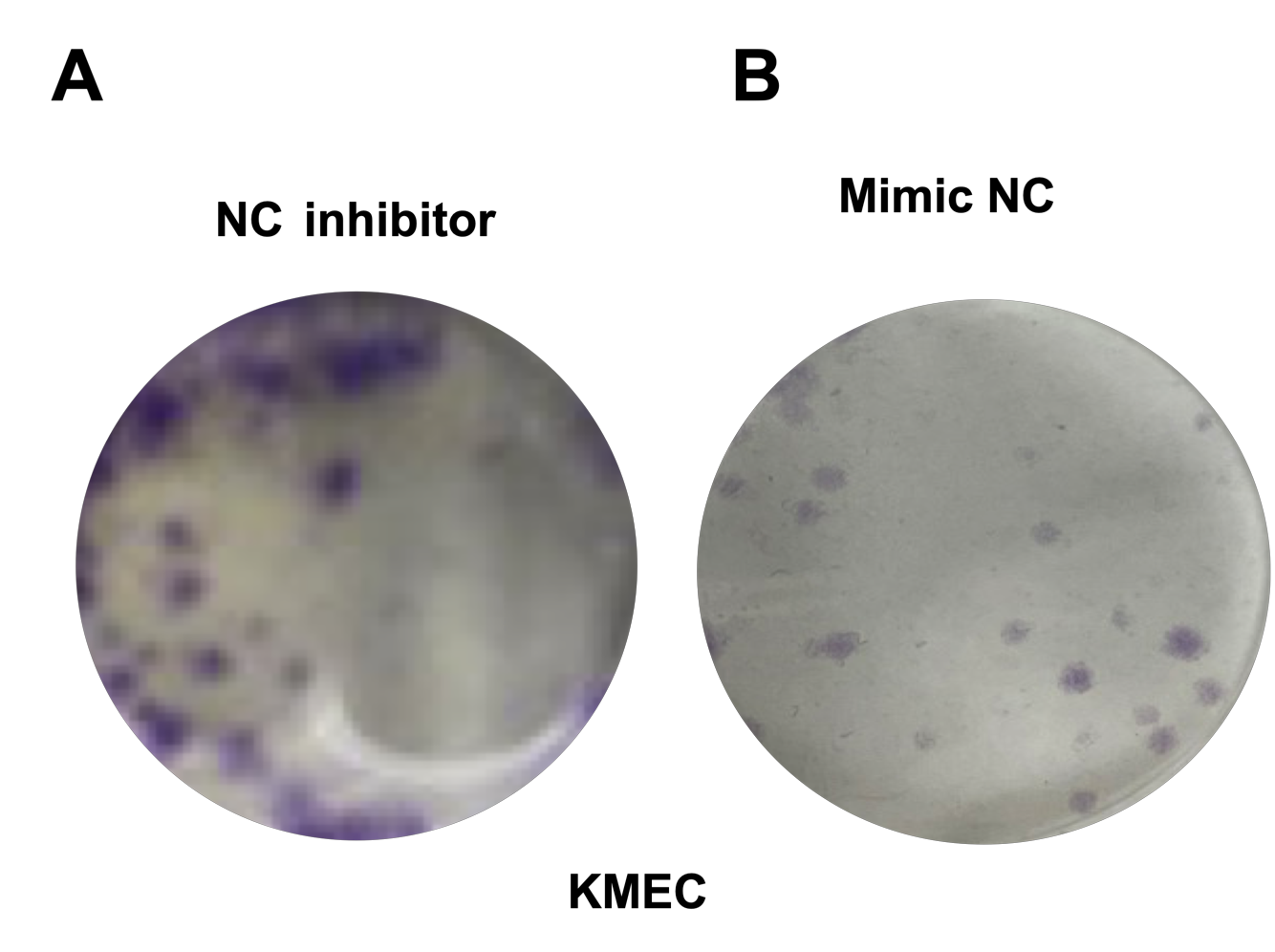


**Supplementary Fig. 2**


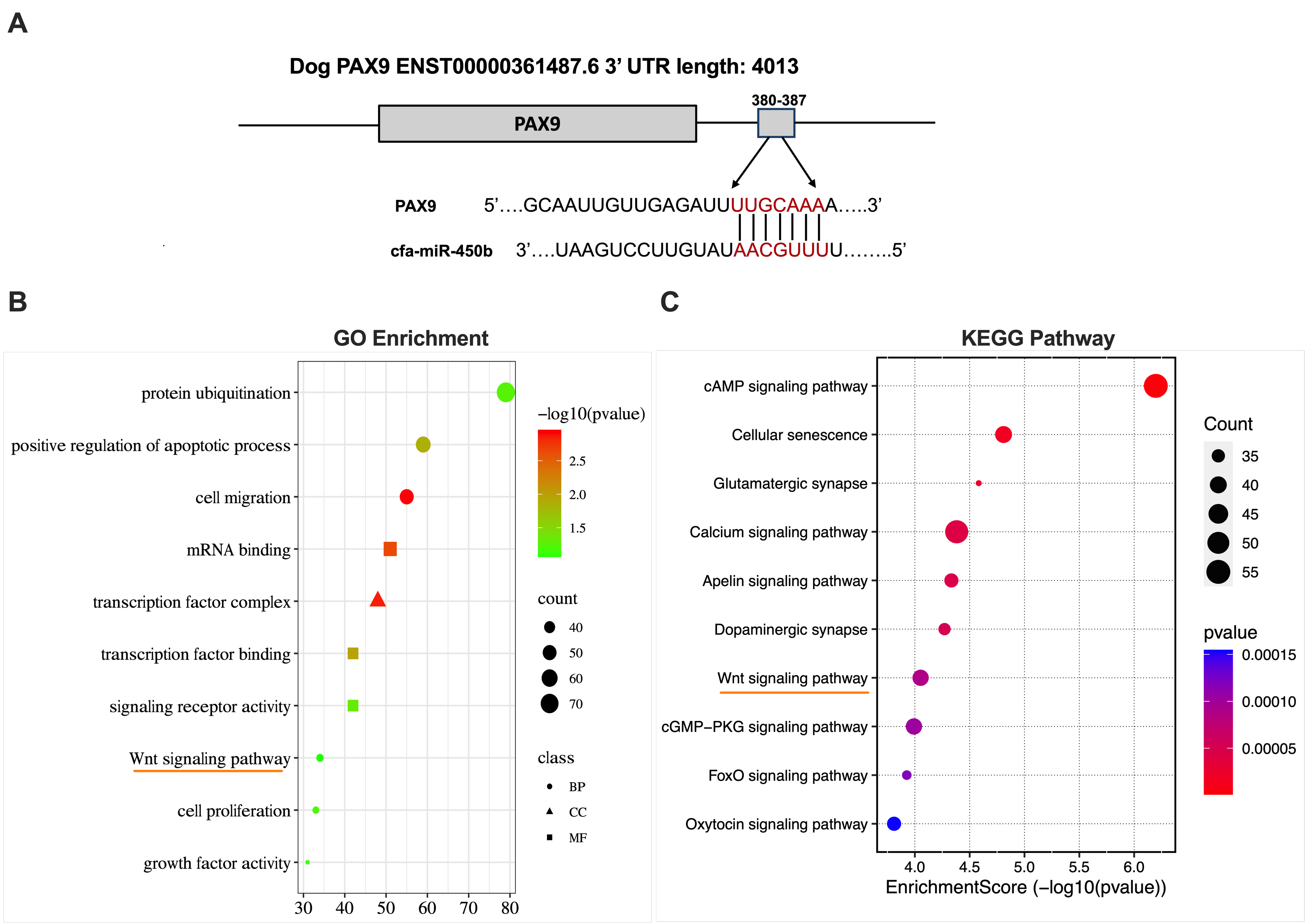


**Supplementary Fig. 3**


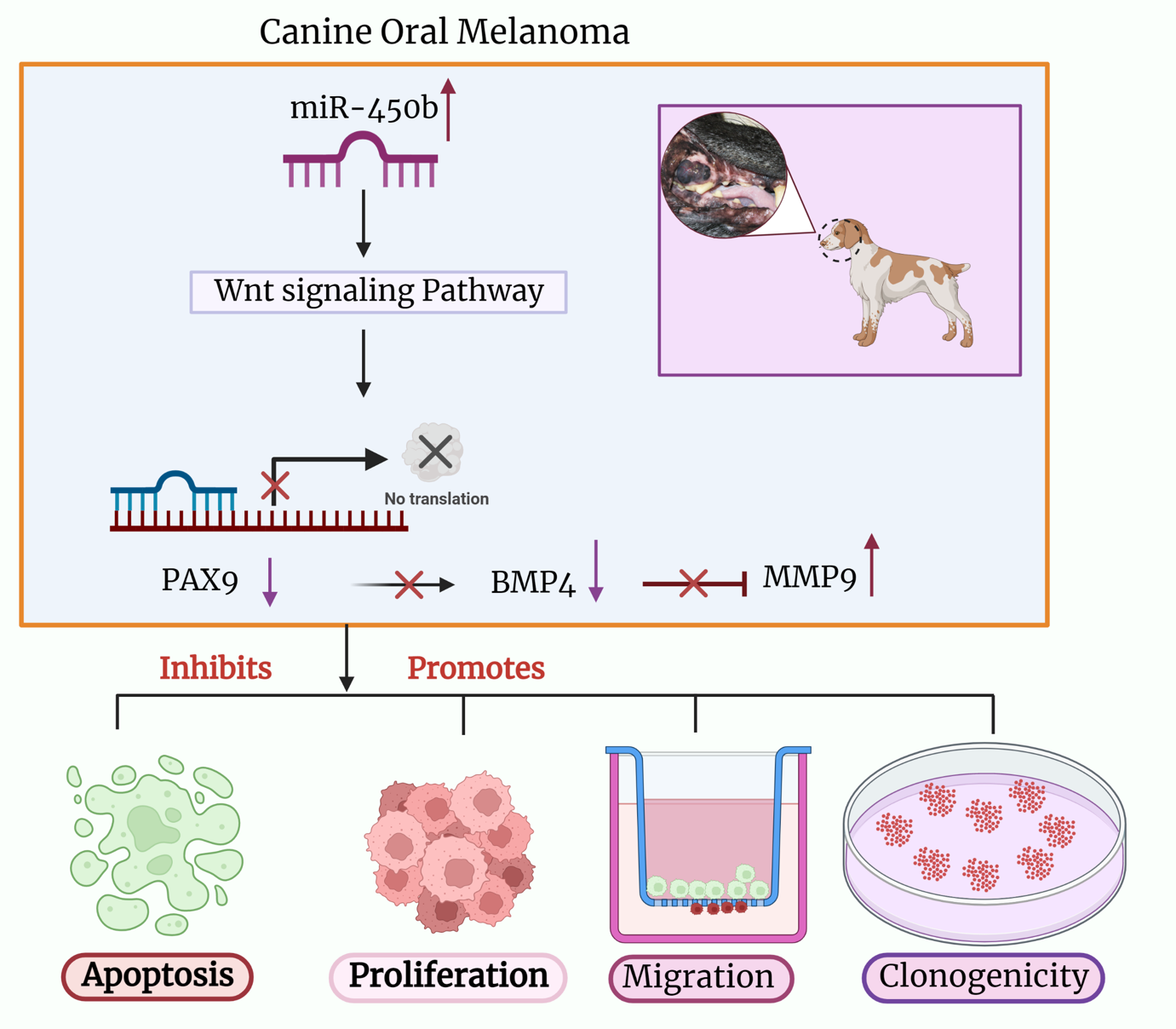


**Supplementary Fig. 4**
